# Oxygen-dependent regulation of serotonin receptor *5-HT*_*1A*_ in hypoxic response and flight performance in bumblebees, *Bombus terrestris*

**DOI:** 10.1016/j.isci.2025.114211

**Published:** 2025-11-25

**Authors:** Chunyan Jiang, Panlong Meng, Xuexiao Du, Yingmin Sun, Xianliang Huang, Bing Chen

**Affiliations:** 1 College of Life Sciences, Hebei University, Baoding, China; 2 Hebei Basic Science Center for Biotic Interaction, Hebei University, Baoding, China

**Keywords:** entomology, biochemistry

## Abstract

Bumblebees, as indispensable pollinators, possess energy-intensive flight that depends on efficient oxygen and energy utilization. However, how aerobic and anaerobic metabolisms in flight muscles are coordinated under varying oxygen levels remains unresolved. Here, we identify the serotonin receptor *5-HT*_*1A*_ as a potential oxygen-responsive factor in the flight muscles of *Bombus terrestris*. Under oxygen-limited conditions (1.6–12 kPa), bumblebees significantly reduced thoracic temperature, respiration, and flight performance and even induced stupor, accompanied by mitochondrial damage in the flight muscles. Transcriptomic analysis under hypoxic conditions revealed 711 differentially expressed genes enriched in G-protein-coupled receptors and energy metabolism pathways. Notably, *5-HT*_*1A*_ expression was upregulated by hypoxia and downregulated by flight activity. Antagonist inhibition of *5-HT*_*1A*_ enhanced flight speed, duration, and distance under both normoxic and hypoxic conditions. Together, these findings suggest that *5-HT*_*1A*_ may mediate oxygen-dependent regulation of flight performance, potentially acting as a negative modulator.

## Highlights

• Hypoxia compromises metabolic function and flight performance in bumblebee flight muscles
• The serotonin receptor *5-HT*_*1A*_ is upregulated by hypoxia
• *5-HT*_*1A*_ is downregulated by flight, and inhibition of *5-HT*_*1A*_ enhances flight ability
• *5-HT*_*1A*_ acts as a potential oxygen-responsive factor in bumblebee flight muscles

## Graphical abstract

**Figure:**
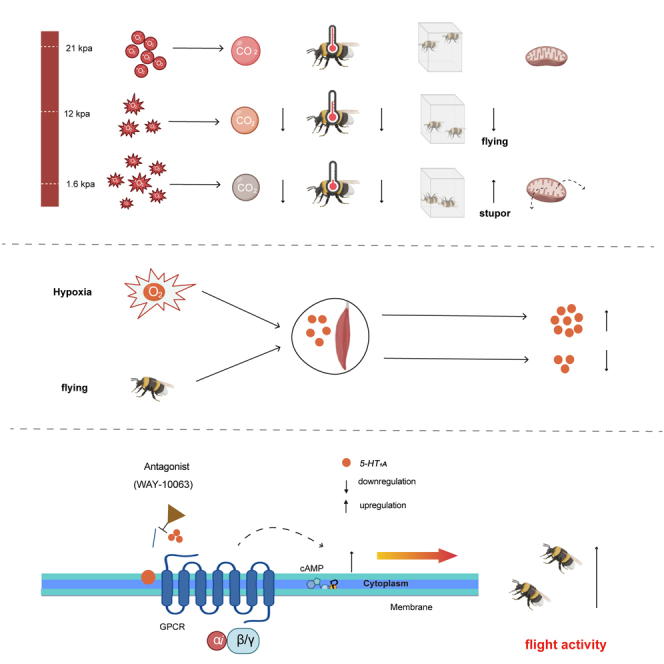

## Introduction

High-altitude hypoxia profoundly impacts the physiology, behavior, and survival of many organisms. However, some insects exhibit remarkable tolerance to low-oxygen conditions. For example, locusts can survive for over 8 h in anaerobic conditions,^1^ and fruit flies can recover from up to 4 h.^2^ Bumblebees, particularly alpine species, frequently experience hypoxic conditions, making them a unique model for studying hypoxia adaptation.^3^^,^^4^ The Qinghai-Tibet Plateau and its surrounding areas, which harbor approximately 50% of the world’s bumblebee species, are characterized by hypoxic environments.^3^^,^^5^^,^^6^^,^^7^ Here, bumblebees have adapted to high altitudes, with many species thriving at elevations above 3,000 m and some even flying at over 4,000 m above sea level.^3^^,^^6^^,^^8^ These adaptations include unique morphological traits, such as variations in body size and wing length,^9^^,^^10^^,^^11^^,^^12^ as well as physiological adjustments in thorax temperature regulation and respiration.^13^ However, research on the physiological and molecular mechanisms underlying oxygen-dependent responses in bumblebees remains relatively limited.^14^

Bumblebees are also pivotal pollinators, playing an indispensable role in both natural and agricultural ecosystems.^15^^,^^16^ However, they have been significantly affected by climate change, which alters their food sources and nesting habitats.^17^^,^^18^^,^^19^ This makes it crucial to understand how bumblebees adapt to environmental stressors, particularly hypoxia. Notably, bumblebees exhibit remarkable flight endurance, covering distances up to 8,000 m daily in search of pollen and nectar.^20^ During foraging, their metabolic rate can increase by 10–100 times compared to their resting state,^21^ with oxygen consumption rising up to 30-fold during flight.^22^^,^^23^ Given the high energy demands of flight, ensuring sufficient oxygen supply and optimal ATP production is critical for their survival. Bumblebees primarily rely on aerobic metabolism fueled by carbohydrates, and alternative energy sources, such as proline, also contribute to sustained flight activity.^24^^,^^25^^,^^26^ While this metabolic strategy is efficient under normal conditions, it raises questions about how bumblebees manage their energy balance for flying activity during periods of reduced oxygen availability.

Energy metabolism regulation is critical for maintaining the balance between aerobic and anaerobic processes, ensuring efficient oxygen utilization and ATP production.^27^^,^^28^ Under aerobic conditions, metabolic regulators optimize oxidative metabolism and mitochondrial function to maximize ATP generation. In contrast, during hypoxia or anaerobic stress, these regulators facilitate a shift to anaerobic pathways, enabling energy production despite limited oxygen availability. This dynamic metabolic flexibility is essential for organisms to adapt to fluctuating environmental conditions and sustain cellular function and survival. A key oxygen-responsive factor, hypoxia-inducible factor (HIF), is degraded by prolyl hydroxylases under normoxia, whereas this degradation is inhibited under hypoxic conditions, resulting in its accumulation and the activation of adaptive transcriptional responses.^27^^,^^29^^,^^30^ However, research on the mechanisms of oxygen-related regulation is relatively limited. Addressing this knowledge gap is essential for understanding how bumblebees cope with high-altitude hypoxic environments and sustain energetically demanding behaviors such as flight.

Here, we explored the effects of different levels of oxygen exposure on *Bombus terrestris* (Linnaeus), a species extensively studied and artificially domesticated for research. We first determined the physiological responses of bumblebees in response to hypoxic stress, particularly focusing on flight activity. Subsequently, we conducted transcriptomic analyses of flight muscles exposed to hypoxia, identifying genes involved in the regulation of oxygen-sensing processes, pinpointing the serotonin receptor *5-HT*_*1A*_, as a potential modulator. Finally, we assessed the negative regulatory role of *5-HT*_*1A*_ in aerobic flight by injecting an antagonist. Overall, these findings reveal a novel regulator involved in the response to oxygen availability.

## Results

### Hypoxic responses in bumblebee physiology, mitochondrial structure, and flight

**Figure 1:**
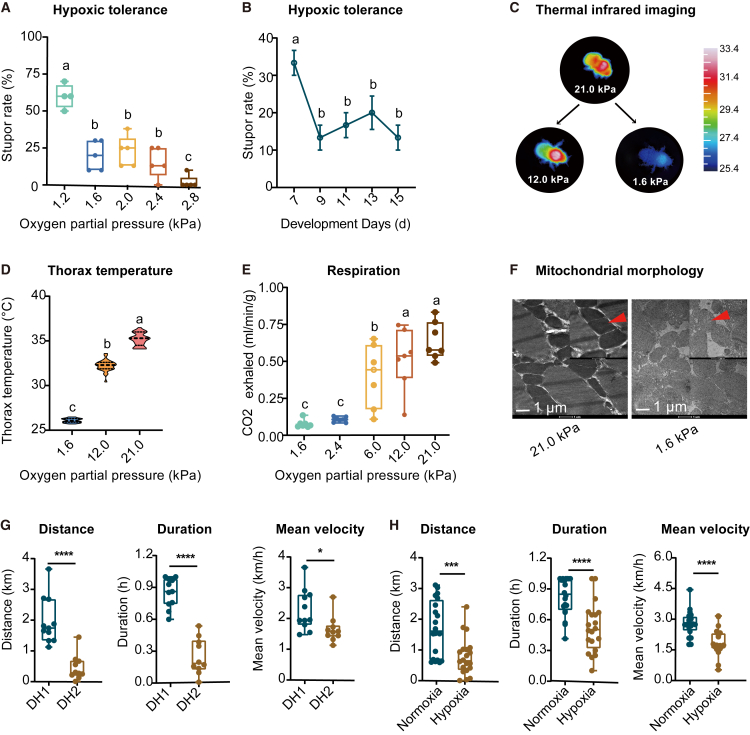
Physiological and behavioral responses of bumblebee workers to mild and extreme hypoxia (A) Stupor rate following various *P*o_2_ treatments for 6 h. *n* = 5 biological replicates of 10 bumblebees for the assays. (B) Stupor rate of bumblebees at different developmental days after 6-h exposure to 1.6 kPa *P*o_2_. *n* = 3 biological replicates of 10 bumblebees in each assay. Distinct letters indicate statistically significant differences (*p* < 0.05), as determined by one-way ANOVA. (C) Respiratory metabolic rates under different *P*o_2_ (*n* = 7). (D) Representative micrographs showing mitochondrial morphology in ultrathin sections of isolated mitochondrial pellets from flight muscles under normoxic (21.0 kPa) and extreme hypoxic (1.6 kPa) conditions. Red arrows indicate mitochondrial cristae. (E) Thermal infrared images displaying thoracic surface temperature. (F) Thoracic surface temperature of bumblebees under different ambient *P*o_2_ levels. (G) Flight performance, including flight distance, duration, and mean velocity, measured under normoxic conditions across different time intervals (*n* = 10). DH1 represents the first hour, and DH2 represents the second hour of the total 2-h measurement. (H) Flight performance under normoxic (21.0 kPa) and mild hypoxic (12.0 kPa) environments (*n* = 20). Data are presented as means (central lines) ± SEM (upper and lower bound of the bars). Statistical significance was determined using Student’s *t* tests.

To elucidate the physiological responses of bumblebees to ambient hypoxia, we initially assessed the tolerance of 13-day-old workers by exposing them to a range of extreme hypoxic conditions, spanning from 2.8 to 1.2 kPa partial oxygen pressure (*P*o_2_) for 6 h (Figure 1A). The stupor rate was 2% at 2.8 kPa, but it significantly increased to 15% at 2.4 kPa and remained stable between 1.6 and 2.4 kPa (one-way ANOVA, F(4, 19) = 24.66, *p* = 2.69E−7). Notably, at 1.2 kPa, the stupor rate rose sharply to 60%, nearly tripling compared to the rate at 1.6 kPa, suggesting that 1.6 kPa may represent a physiological threshold for sustaining metabolic activity. Accordingly, 1.6 kPa was selected as the extreme hypoxia condition for subsequent experiments.

We then investigated developmental variation in hypoxia tolerance. At 1.6 kPa *P*_O2_, 7-day-old adults exhibited a higher stupor rate of 33%, whereas it stabilized between 13% and 20% from day 9 to day 15 post eclosion (one-way ANOVA, F(4, 12) = 3.72, *p* = 0.034, Figure 1B). Considering that workers aged 7–13 days also demonstrated stronger flight performance than younger foragers (Figure S1), we selected 13-day-old workers for the following experiments.

To assess thermoregulatory responses under hypoxia, thoracic surface temperature was measured using infrared thermal imaging (Figure 1C). The thoracic temperature gradually decreased as oxygen availability declined, from 35.1°C at 21 kPa to 32.2°C at 12.0 kPa and down to 26.1°C at 1.6 kPa (Figure 1D). The results indicate that mild hypoxia induced metabolic suppression, whereas extreme hypoxia almost halted thermogenic activity.

Respirometry measurements revealed a dose-dependent suppression of metabolic rate under decreasing oxygen levels (Figure 1E). At 12.0 kPa, CO_2_ emissions remained unchanged compared to normoxic conditions. However, exposure to 6.0 kPa significantly reduced CO_2_ emission (*p* = 0.016). Under severe hypoxia (2.4 and 1.6 kPa *P*o_2_), the metabolic rate dropped by over 82%, from 0.64 to 0.11 and 0.08 mL min^−1^ g^−1^, respectively. These findings suggest that the respiration of bumblebee is robust to moderate oxygen limitation but is markedly suppressed under extreme hypoxia. Correspondingly, ultrastructural analysis of mitochondria in flight muscle revealed severe morphological disruptions under extreme hypoxia, including swelling and cristae disorganization (Figure 1F).

To evaluate the effects of hypoxia on flight capability, we conducted tethered-flight assays measuring flight distance, duration, and mean velocity. Bumblebees exhibited a much stronger flight capacity during the first hour compared to the second hour. Thus, the first-hour duration was selected for subsequent tests (Figure 1G). Mild hypoxia (12 kPa *P*o_2_) significantly impaired flight performance compared to normoxia (Figure 1H). Specifically, flight distance of workers decreased by 58% (Student’s *t* test, *p* = 3.56E−8), duration by 37% (*p* = 5.80E−5), and mean velocity by 32% (*p* = 7.57E−5) upon hypoxic exposure. Evidently, hypoxia substantially suppresses the flight capacity of bumblebees.

In summary, hypoxia exerts prominent effects on bumblebees: severe hypoxic stress induces stupor, suppresses respiration and thermoregulation, and impairs mitochondrial morphology, while mild hypoxia reduces respiratory metabolism, lowers body temperature, and diminishes flight capacity.

### Transcriptomic profiling revealed major pathways triggered by extreme hypoxia in flight muscle

**Figure 2:**
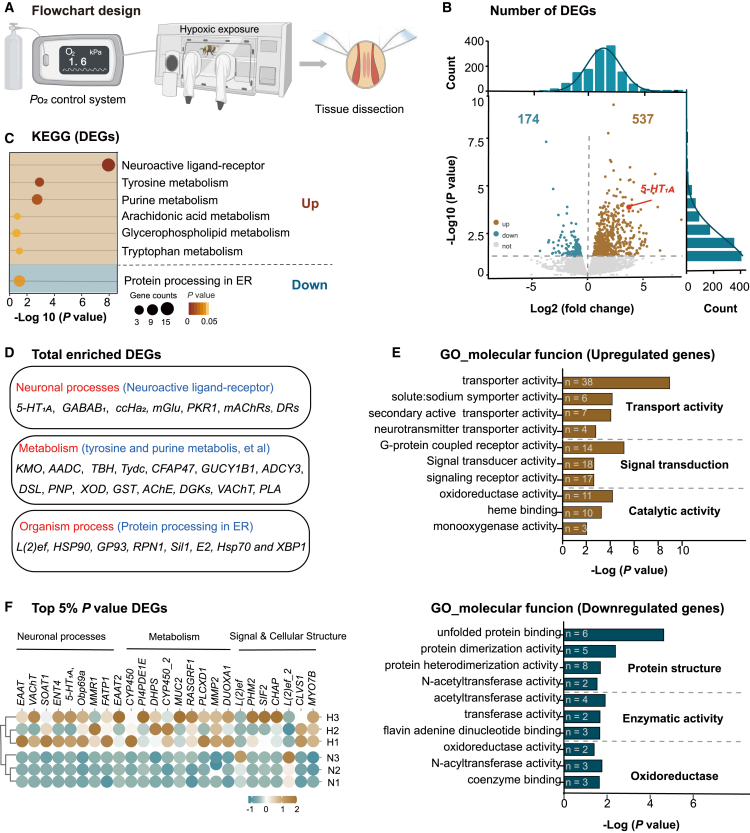
Transcriptomic responses of bumblebee flight muscles under extreme hypoxia (A) Experimental workflow for hypoxic exposure (1.6 kPa) and transcriptomic analysis. (B) Volcano plot of differentially expressed genes (DEGs) under hypoxic conditions. Downregulated genes are highlighted in blue, upregulated DEGs in caramel, and non-significant genes in gray. (C) KEGG pathway enrichment analysis of DEGs responsive to hypoxia (*p* < 0.05). (D) Functional classification of all significantly enriched DEGs. (E) GO analysis of molecular functions for downregulated and upregulated DEGs. The most significantly enriched terms (*p* < 0.05) are shown. (F) Heatmap showing the top 5% most significant DEGs (*p* < 0.05).

To elucidate the molecular mechanisms by which hypoxia impairs the flight activity in foragers, we performed transcriptome sequencing on the flight muscles of bumblebees following a 6-h exposure to 1.6 kPa *P*o_2_ (Figure 2A). A total of 711 differentially expressed genes (DEGs) were identified (*p* value < 0.05), including 537 upregulated and 174 downregulated genes (Figure 2B). These findings indicate a predominant upregulation of gene expression in response to hypoxic stress in the flight muscles of bumblebees.

Subsequently, Kyoto Encyclopedia of Genes and Genomes (KEGG) and Gene Ontology (GO) enrichment analyses were performed on these DEGs. The upregulated DEGs were predominantly involved in neural signaling pathways (Figure 2C), such as neuroactive ligand-receptor interactions (*p* = 1.21E−8). Eight genes with functional annotations related to neuronal processes exhibited significant upregulation in response to hypoxia in bumblebees (Figure 2D). These genes included *5-HT*_*1A*_, *GABAB1*, *CCHa2 mGlu*, and *PKR1*, which are involved in modulating synaptic activity and regulating neurotransmitter release^31^^,^^32^; *GRIN*, which encodes the NMDA receptor involved in synaptic plasticity, learning, and memory^33^^,^^34^; and *mAChRs* and *DRs*, which contribute to olfactory aversive learning and memory in *Drosophila*.^35^^,^^36^^,^^37^ Additional significantly enriched pathways were associated with metabolism, including tyrosine metabolism (*p* = 1.10E−3), purine metabolism (*p* = 1.68E−3), arachidonic acid metabolism (*p* = 0.03), glycerophospholipid metabolism (*p* = 0.03), and tryptophan metabolism (*p* = 0.03). These pathways involved genes such as *KMO*, which plays a role in cellular energy production and tryptophan catabolism; *AADC*, *TBH*, *Tydc*, and *CFAP47* genes, which are related to tyrosine metabolism; *PDE*, *GUCY1B1*, *ADCY3*, *ADSL*, *PNP*, and *XOD*, which are associated with purine nucleotides involved in energy production and signal transduction; *GST*, which is involved in regulating inflammation; and *AChE*, *DGKs*, *VAChT*, and *PLA*, which are linked to lipid metabolism and signaling regulation. In contrast, the downregulated DEGs were primarily significantly enriched in protein processing within the endoplasmic reticulum (*p* = 3.01E−5). These genes, which participate in protein folding, quality control, and stress response, included *L(2)ef*, *HSP90*, *GP93*, *RPN1*, *Sil1*, *E2*, *Hsp70*, and *XBP1*. GO enrichment analysis of molecular function terms revealed that downregulated genes were predominantly associated with protein structure, enzymatic activity, and oxidoreductase function, while upregulated genes were enriched in transport activity, signal transduction, and catalytic activity (Figure 2E).

To further identify key candidate genes involved in hypoxic response, we identified 25 DEGs based on the top 5% of the lowest *p* values (Figure 2F). These DEGs clustered into three major functional categories: neuronal processes, metabolic pathways, and signal and cellular structure. Among them, the majority were associated with neuronal function, followed by genes involved in metabolic regulation.

Analysis of DEGs revealed that neuro-signaling-related pathways were among the top enriched categories in KEGG. They were also represented within the top 5% of functional categories ranked by statistical significance. This pattern indicates that peripheral neural signaling may play an important role in the hypoxia response. Among these, 5-hydroxytryptamine receptor was identified as a key candidate, exhibiting a high fold change and statistically significant differential expression.

In summary, genes associated with neuronal signaling and metabolic processes could play key roles in mediating the response to hypoxic stress.

### Validation of neuroactive and carbohydrate metabolic pathways in the hypoxia response

**Figure 3:**
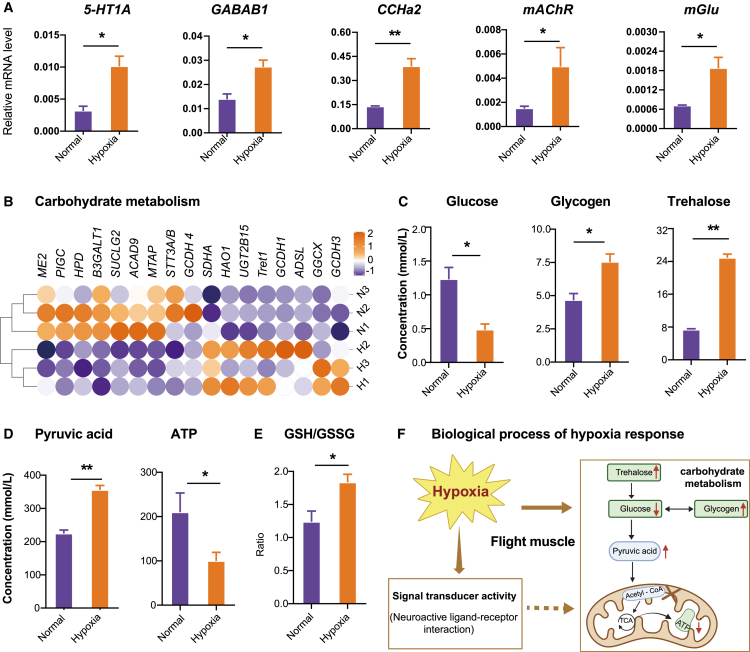
Neuroactive and carbohydrate metabolic pathways in flight muscles under extreme hypoxia in *B. terrestris* (A) Transcriptome validation of randomly selected key genes (e.g., *5-HT*_*1A*_, etc.) in the neuroactive ligand-receptor interaction pathway using quantitative reverse-transcription PCR (RT-qPCR). *n* = 3 biological replicates of three bumblebees for all assays. (B) Expression profiles of genes involved in sugar metabolism pathways. Heatmap showing log_2_(fold change) in response to hypoxic exposure. N1–3 represent the control group, and H1–3 represent the hypoxic treatment group. (C) Measurement of glucose, glycogen, and trehalose concentrations involved in carbohydrate metabolism. (D) Quantification of pyruvate and ATP levels. (E) Measurement of the GSH/GSSG ratio as an indicator of cellular redox status. *n* = 3 biological replicates of three bumblebees for all assays. Data are presented as means ± SEM. Statistical significance between groups was determined using Student’s *t* tests. (F) Proposed model of hypoxia-regulated biological processes in flight muscles, integrating transcriptomic and targeted metabolite quantification.

We next assessed the mRNA levels of key DEGs associated with neuroactive receptors (Figure 3A). The results showed that all of these genes exhibited differential expression pattern consistent with the transcriptomic data. Notably, *5-HT*_*1A*_ has been previously reported to play a crucial role in metabolic and behavioral regulation.^38^^,^^39^^,^^40^ Expression analysis revealed that *5-HT*_*1A*_ mRNA levels in flight muscles were significantly increased by 3.2-fold following hypoxia exposure (*p* = 0.017).

We then validated metabolic changes in the flight muscle. Expression profiling revealed substantial alterations in the expression of genes involved in carbohydrate metabolism in response to hypoxic exposure (Figure 3B). Severe hypoxia significantly decreased glucose levels (Student’s *t* test, *p* = 0.019, *n* = 3), while glycogen (*p* = 0.022, *n* = 3) and trehalose levels (*p* = 0.003, Figure 3C) were significantly elevated. Compared to control conditions, hypoxia led to a significant reduction in ATP concentration and an increase in pyruvic acid levels (pyruvic acid, *p* = 0.002; ATP, *p* = 0.016; *n* = 3, Figure 3D). Furthermore, antioxidant capacity was maintained, as indicated by a high ratio of glutathione (GSH) to oxidized glutathione (GSSG) (*p* = 0.040, *n* = 3, Figure 3E).

Taken together, these results suggest that extreme hypoxia induces coordinated physiological responses in bumblebees, including the activation of neuroactive signaling pathways, significant remodeling of carbohydrate metabolism, and enhancement of antioxidant defenses (Figure 3F). Notably, the upregulation of *5-HT*_*1A*_ suggests its potential role in linking neural signaling with metabolic and behavioral responses under hypoxia.

### Regulation of *5-HT*_*1A*_ expression in response to hypoxia and aerobic flight

**Figure 4:**
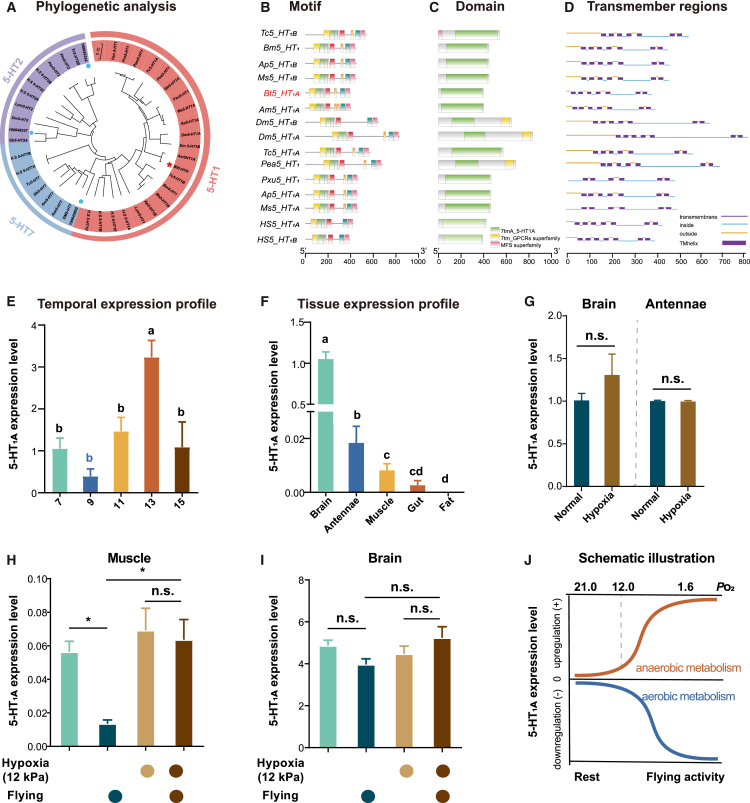
Structure and expression analysis of the *5-HT*_*1A*_ gene in relation to development, hypoxia, and flight *n* = 5 biological replicates of three bumblebees for all assays (A) Phylogenetic relationships of *5-HT* family genes from representative mammals and insects, analyzed with 1,000 bootstrap replicates in MEGA7. (B–D) Characterization of motifs, domains, and predicted transmembrane regions in 5-HT1 proteins. Scale bars represent gene length. (E) Expression levels of the *5-HT*_*1A*_ gene at different developmental days, measured by quantitative real-time PCR. The *UBI* gene was used as an internal control. (F) Expression levels of the *5-HT*_*1A*_ gene in various tissues under normoxic conditions. (G) Gene expression levels in the brain (left) and antennae (right) following hypoxic exposure. (H and I) Gene expression levels in flight muscle and brain following mild hypoxic exposure and flight treatments. Four biological replicates of 13-day-old workers were used for each treatment. (J) Schematic illustration depicting alterations in *5-HT*_*1A*_ gene expression during aerobic and anaerobic metabolic processes. Data are presented as means ± SEM. Different letters indicate significant differences (*p* < 0.05) as determined by one-way ANOVA. Statistical significance: ∗*p* < 0.05, n.s., no significance (Student’s *t* test).

To investigate the evolutionary relationships of 5-HT receptor genes, we constructed a phylogenetic tree using amino acid sequences from various species. The 5-HTR gene in *B. terrestris* was found to be most closely related to the *5-HT*_*1A*_ gene of two other insect species, *Apis mellifera* (Figure 4A; Table S2), supporting its classification as *5-HT*_*1A*_.

We further analyzed the gene structures of 5-HT1 in 16 species using MEME software and identified seven conserved motifs, designated as motif 1–7 (Figure 4B). Except for motif 5, these motifs were highly conserved across 5-HT1 protein sequences. Notably, motif 5 was absent in mammalian *5-HT*_*1A*_ and *5-HT*_*1B*_ receptors but present in all insects *5-HT*_*1A*_ sequences examined. Conserved domains, such as 7tmA, also displayed strong conservation (Figure 4C). In addition, transmembrane domain analysis using the TMHMM database revealed that the *B. terrestris 5-HT*_*1A*_ protein contains seven transmembrane helices, a characteristic typical of G-protein-coupled receptors (Figure 4D), consistent with known receptor structures.

To explore developmental and tissue-specific expression patterns of *5-HT*_*1A*_ under normoxic conditions, we analyzed its expression across different stages and tissues. *5-HT*_*1A*_ expression peaked on the 13th day after adult emergence (Figure 4E). Among tissues, expression was highest in the brain, followed by the antennae, flight muscle, and gut, with minimal expression in the fat body (Figure 4F). We then examined expression changes in the brain and antennae under hypoxia (Figure 4G). In contrast to the differential *5HT*_*1A*_ expression observed in flight muscles, neither the brain nor the antennae exhibited significant changes in *5HT*_*1A*_ expression following hypoxic treatment. These findings indicate that *5-HT*_*1A*_ expression is regulated in a developmental and tissue-specific manner^41^ and is modulated by hypoxia only in specific tissues, such as the flight muscle.

To explore the functional relevance of *5HT*_*1A*_ expression in flight muscle, we examined its regulation by flight activity under different oxygen conditions. Under normoxic flight conditions, *5-HT*_*1A*_ expression in flight muscles was significantly downregulated after 1-h sustained flying compared to the resting stage (Figure 4H). However, under mild hypoxia, flight activity no longer had any effect on *5-HT*_*1A*_ expression. Notably, under flying condition, even mild hypoxia significantly upregulated *5-HT*_*1A*_ expression. In contrast, no significant changes in *5-HT*_*1A*_ expression were observed in brain tissues in response to either hypoxia or flying (Figure 4I). Specifically, under flight conditions, *5-HT*_*1A*_ expression in the flight muscles of the hypoxia-treated group was significantly higher than that in the normoxic group, while brain tissue showed no detectable alterations. Moreover, the expression of other serotonin receptor subtypes (*5-HT*_*2A*_, *5-HT*_*2B*_, and *5-HT*_*7*_) in flight muscles remained unchanged under extreme hypoxia (Figure S2).

To summarize, hypoxia and flying exert distinct and oxygen-dependent effects on *5-HT*_*1A*_ expression in flight muscle. Specifically, extreme hypoxia induces upregulation of *5-HT*_*1A*_ expression while flight suppresses its expression (Figure 4J).

### *5-HT*_*1A*_ exerts an inhibitory effect on flight activity

**Figure 5:**
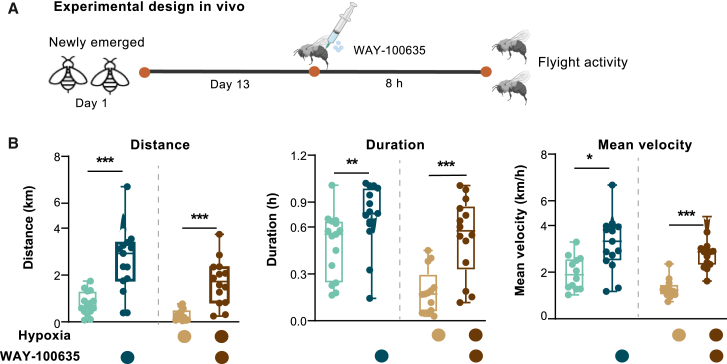
Effect of *5-HT*_*1A*_ inhibition on aerobic flight activity under normal and hypoxic conditions in *B. terrestris* (A) Experimental design for *in vivo* functional validation of *5-HT*_*1A*_. *5-HT*_*1A*_ activity was inhibited by injection of *5-HT*_*1A*_ antagonist, WAY-100635. (B) Effects of *5-HT*_*1A*_ antagonist supplementation on flight performance under both normoxic (21 kPa) and hypoxic (12 kPa) conditions. *n* = 15 workers were assessed under all conditions. Data are presented as means ± SEM. Statistical significance was determined using Student’s *t* test: ∗*p* < 0.05, ∗∗*p* < 0.01, ∗∗∗*p* < 0.001, ∗∗∗∗*p* < 0.0001.

To elucidate the specific contribution of *5-HT*_*1A*_ to regulating bumblebee flight performance, a 1-h sustained flight assay was performed on bumblebees supplemented with the potent and selective *5-HT*_*1A*_ antagonist, WAY-100635 (Figure 5A). Under normoxic conditions, treatment with the antagonist significantly increased flight distance by 253% (Student’s *t* test, *p* = 1.08E−4, *n* = 15, Figure 5B). This enhancement was even more pronounced under hypoxic conditions, reaching a 448% increase (*p* = 4.04E−5). Flight duration was also significantly extended. Under normoxic conditions, it was prolonged by 54% (*p* = 6.00E−3), and under hypoxic conditions, by 216% (*p* = 1.78E−4), compared to the control group. Regarding mean velocity, a significant increase was observed as well: from 1.65 to 3.40 km/h under normoxia (*p* = 3.83E−4) and from 1.55 to 2.80 km/h under hypoxia (*p* = 6.51E−5). Collectively, these findings strongly indicate that *5-HT*_*1A*_ plays an inhibitory role in regulating bumblebee flight performance.

## Discussion

In this study, we quantified oxygen-sensing ability in bumblebees and explored the effects of hypoxia on flight activity and the underlying mechanisms using physiological, transcriptomic, and functional analyses. Bumblebees are subjected to stress from both ambient hypoxia, which induces anaerobic conditions, and the aerobic demands of foraging flight. In response to these challenges, they employ a coordinated regulatory strategy to balance aerobic and anaerobic processes, ensuring long-term survival and reproductive success. The study provides the first evidence that the serotonin receptor *5-HT*_*1A*_ in flight muscle plays a potential key role in modulating energy conversion in response to oxygen levels. This study of *B. terrestris* provides novel insights into the mechanisms of oxygen-related metabolism in bumblebees.

Bumblebees exhibit a remarkable tolerance to hypoxia compared to mammals.^42^ For instance, most bumblebees remain active under severe hypoxic exposure at 2.8 kPa *P*o_2_, showing a low incidence of stupor, similar to the Tibetan locust.^43^ Hypoxia at varying degrees significantly impacts the physiological performance of bumblebees, manifesting as reduced respiratory metabolic rates, altered mitochondrial morphology under extreme hypoxia, and impaired flight capacity under moderate hypoxia. Extensive research has confirmed the physiological impacts of hypoxia in various insect species. Severe hypoxia (1–5 kPa *P*o_2_), as observed in *Drosophila melanogaster* and *Locusta migratoria*,^43^^,^^44^ significantly reduced metabolic rates and decreased the activity of the mitochondrial complex cytochrome *c* oxidoreductase, thereby limiting the ability to meet metabolic demands. Mild hypoxia (5–15 kPa *P*o_2_) has been shown to suppress essential behaviors, including feeding,^45^ flight,^46^ and other vital activities.^47^ Given the muscle-powered foraging flight of bumblebees, varying levels of hypoxia induce specific changes in muscle metabolism and contractility. The severity of hypoxic stress affects these processes accordingly, enabling bumblebees to adjust energy consumption and sustain flight in hypoxic environments, such as mountainous regions.^48^ Together, these findings suggest that the bumblebee *B. terrestris* has changes of physiological and behavioral strategies with decreasing oxygen levels.

To elucidate the mechanisms underlying the detrimental effects of hypoxia, we combined transcriptomic analyses with physiological and biochemical assays in bumblebees. Further transcriptomic analysis revealed that a substantial number of DEGs were influenced by hypoxia. Notably, DEGs associated with neural activation and transport pathways were strongly enriched in extreme hypoxia, suggesting a significant role in the anaerobic process. In contrast, gene expression related to protein processing in the endoplasmic reticulum was significantly suppressed, corroborating studies that show disrupted protein folding and aggregation under hypoxic stress.^49^^,^^50^ Moreover, the energy requirements for sustaining daily activities (i.e., mating, foraging, and predator evasion) become even more critical under hypoxic stress. Carbohydrates, as the primary fuel source for bumblebee survival, undergo significant changes during anaerobic processes. Overall, anaerobic conditions trigger the activation of neural genes (e.g., *5-HT*_*1A*_ signaling), which may influence carbohydrate metabolism and suppress ATP production, thereby optimizing energy allocation for colony survival.

The study further identified the serotonin receptor *5-HT*_*1A*_ as a regulator of both anaerobic and aerobic metabolic processes. Typically, 5-HT exerts its effects by selectively binding to specific 5-HT receptors, modulating various physiological functions, including aggression, movement control, and thermoregulation in both animals and humans.^51^^,^^52^^,^^53^^,^^54^^,^^55^^,^^56^^,^^57^^,^^58^^,^^59^ While 5-HT receptors are classified into seven types in vertebrates,^60^^,^^61^ insects have 5-HT_1_, 5-HT_2_, and 5-HT_7_ receptor subtypes based on sequence similarities and the activation of second-messenger pathways.^62^^,^^63^^,^^64^ Within the serotonin receptor family, we found that 5-HT_1A_ was the only subtype exhibiting an anaerobic transcriptional response, which was tissue specificity, and it also displayed differential expression during flight. The role of *5-HT*_*1A*_ has been studied in species, such as *Drosophila melanogaster* and *Apis mellifera*,^53^^,^^65^^,^^66^^,^^67^^,^^68^ but its role in regulating flight performance remains unexplored.

Here, we propose that *5-HT*_*1A*_ acts downstream in a cascade of pathways that regulate flight processes. Flight, a complex aerobic behavior, requires precise coordination of sensory and neuroendocrine control, efficient energy metabolism, and muscle contraction.^69^^,^^70^^,^^71^^,^^72^^,^^73^ Given the substantial energy consumption during foraging, disturbances in energy-related metabolic processes can significantly impact flight capacity. Many functional molecules involved in regulating flight have been identified, including the HIF *Hif-1α2*, which has been extensively reported in numerous animals^74^ and is known to facilitate prolonged flight performance in migratory locust.^75^ Some complex neuroendocrine networks also play a crucial role in regulating tissue-specific metabolism during flight. For instance, the adipokinetic hormone and corazonin-related peptide have been shown to be conserved regulators of flight-related energy metabolism across various insect species.^61^^,^^76^^,^^77^^,^^78^ Other neuromodulators, such as serotonin and octopamine, are also critical in modulating the complex processes involved in flight.^79^^,^^80^ Serotonin, as a general modulator, inhibits motor behavior, including reducing flight motor output in moths,^81^ slowing walking speed in flies,^82^ and decreasing social aggression and disordering appetite and sleep patterns.^83^ In this study, we demonstrate that the selective *5-HT*_*1A*_ antagonist WAY-100635 significantly enhances flight activity in tethered bumblebees. Although our study did not directly investigate the cAMP pathway, previous evidence indicates that the underlying mechanisms likely involve alterations in cAMP levels, which, in turn, affect muscle contraction through Ca^2+^ signaling and glucose metabolism, key processes for energy supply during flight.^84^ Moreover, *5-HT*_*1A*_ regulates multiple downstream signaling pathways, including nitric oxide synthase, NADPH oxidases, and various kinases (e.g., mitogen-activated protein kinase, phosphatidylinositol 3-kinase, PLC, and ERK).^85^^,^^86^^,^^87^ These findings suggest that *5-HT*_*1A*_ plays a key role in modulating energy metabolism and activating signaling cascades to regulate flight capacity.

As an important member of the serotonin receptor family, understanding the role of *5-HT*_*1A*_ in bumblebee flight provides new insights into flight regulation and contributes to a broader understanding of the diverse roles of serotonin receptors in insect physiology. Previous studies have shown that serotonin receptors are essential for various biological functions, including movement,^38^^,^^39^^,^^40^ learning and memory,^88^ and sleep regulation.^54^^,^^89^ While previous research has explored the role of *5-HT*_*1A*_ in behaviors, such as phototaxis in bees^90^ and aggression in fruit flies,^91^ the current study is the first to emphasize its role in modulating bumblebee flight, thus advancing understanding of this receptor’s diverse functions in insect behavior. By elucidating *5-HT*_*1A*_-mediated modulation of flight through the cAMP pathway, the research advances the understanding of how receptors contribute to insect behavior, highlighting the novel role of *5-HT*_*1A*_ in regulation of flight dynamics. Our findings highlight a regulatory role of *5-HT*_*1A*_ in oxygen-dependent physiological processes, particularly in response to hypoxia and during flight.

### Limitations of the study

A limitation of this study is the lack of direct functional validation linking *5-HT*_*1A*_ to the regulation of flight performance under different oxygen conditions. Although differential expression of *5-HT*_*1A*_ was observed under flight and extreme hypoxia, the causal role of this receptor in mediating the aerobic-to-anaerobic transition remains unconfirmed. Future studies should incorporate targeted gene knockdown or editing approaches (e.g., RNAi and CRISPR-Cas9) to determine whether *5-HT*_*1A*_ modulates flight via downstream metabolites (e.g., cAMP and energy substrates) or flight control systems (e.g., visual flow and perception). However, such molecular tools are currently limited in bumblebees, highlighting a need for the development of species-specific genetic techniques.

## Resource availability

### Lead contact

Further information and requests for resources should be directed to and will be fulfilled by the lead contact, Prof. Dr. Bing Chen (chenbing@hbu.edu.cn).

### Materials availability

This study did not generate new materials.

### Data and code availability

• The transcriptome sequencing data have been deposited at the China National Center for Bioinformation (CNCB, https://ngdc.cncb.ac.cn/gsa/s/h48YPeP2) under the accession number CRA033245 and will be publicly available as of the date of publication.
• This paper does not report original code.
• Any additional information required to reanalyze the data reported in this paper is available from the lead contact upon request.

## Acknowledgments

We thank Jiaxing Huang for providing bumblebee culturing techniques. The project was supported by the 10.13039/501100001809Natural Science Foundation of China (nos. 32470447 and W2412134), the 10.13039/501100003787Hebei Natural Science Foundation (no. C2022201042).

## Author contributions

Conceptualization, B.C. and C.J.; methodology, C.J. and P.M.; investigation, C.J., P.M., X.D., X.H., and Y.S.; writing – original draft, B.C. and C.J.; writing – review and editing, B.C. and C.J.; funding acquisition, B.C.; resources, B.C.; supervision, B.C.

## Declaration of interests

The authors declare no competing interests.

## STAR★Methods

### Key resources table

**Table:** 

REAGENT or RESOURCE	SOURCE	IDENTIFIER
**Biological samples**		

Flight muscle tissues of *Bombus terrestris*	Zhongnong Fengshou Ecological Agricultural Technology Co., Ltd., China	N/A
Brain, antennae, gut, and fat body tissues of *Bombus terrestris*	Zhongnong Fengshou Ecological Agricultural Technology Co., Ltd., China	N/A

**Chemicals, peptides, and recombinant proteins**		

WAY-100635 (5-HT1A antagonist)	MedChemExpress (MCE), USA	CAS: 162760-96-5
Dimethyl sulfoxide (DMSO)	Sigma-Aldrich	Cat# D2650
Paraformaldehyde (4%)	Solarbio, China	Cat# P1110
Glutaraldehyde (2.5%)	Solarbio, China	Cat# G1102

**Critical commercial assays**		

Pyruvate (PA) assay kit (microplate method)	Solarbio, China	Cat# BC2205
ATP assay kit (microplate method)	Boxbio, China	Cat# AKOP004M
Glycogen assay kit (colorimetric method)	Nanjing Jiancheng, China	Cat# A043-1-1
Glucose assay kit (microplate method)	Nanjing Jiancheng, China	Cat# A154-1-1
Trehalose assay kit (anthrone colorimetric method)	Nanjing Jiancheng, China	Cat# A149-1-1
Glutathione (GSH/GSSG) assay kit (microplate method)	Nanjing Jiancheng, China	Cat# A061-1-1
Eastep® Super Total RNA Extraction Kit	Promega, USA	Cat# LS1040
SYBR Green qPCR Kit	Roche, USA	Cat# 04707516001

**Deposited data**		

RNA-seq data	This paper	Database: CRA033245

**Experimental models: Organisms/strains**		

*Bombus terrestris* colonies	Zhongnong Fengshou Ecological Agricultural Technology Co., Ltd., China	N/A

**Oligonucleotides**		

Primers for qRT-PCR (see Table S1)	This paper	N/A

**Software and algorithms**		

GraphPad Prism 8.0.2	GraphPad Software	N/A
ImageJ	ImageJ	N/A
MEGA 11	MEGA Software	N/A
TBtools	TBtools Software	N/A
LightCycler 480 Software	Roche, USA	N/A
ExpeData software (v1.9.27)	Sable Systems International	N/A
SPSS version 15.0	SPSS Inc., Chicago, IL, USA	N/A
MEME Suite	MEME Suite Server	Introduction - MEME Suite
TMHMM Server v.2.0	CBS, Technical University of Denmark	TMHMM-2.0 - redirect

**Other**		

Modified flight mill system for flight assay	This paper	N/A
Hypoxic chamber (Model: FLYDWC-50)	Fenglei Co., Ltd., China	N/A
Transmission electron microscope (Model: JEM-100SX)	JEOL, Japan	N/A
DESeq2	OmicShare	https://www.omicshare.com/

### Experimental model and study participant details

The experiments were conducted between May 2023 and July 2025 using female worker bumblebees (*Bombus terrestris*) obtained from a commercial supplier (Zhongnong Fengshou Ecological Agricultural Technology Co., Ltd., China). Ten colonies were used in total. Each colony was delivered in a standard plastic nest box (29 × 22.5 × 13 cm) and maintained under controlled laboratory conditions at the Integrated Stress Adaptation Biology Laboratory, College of Life Sciences, Hebei University (Baoding, China). Environmental conditions were kept at 26°C–28°C, 60% relative humidity, and continuous darkness.

Newly emerged adult workers (0–24 h post-emergence; female) were collected daily and transferred to individual plastic boxes (8 × 8 × 11 cm) for subsequent experiments. Colonies were provided daily with fresh pollen candy (a mixture of pollen and 50% sugar solution) and had continuous access to 50% sucrose solution supplied through an external reservoir. Because *B. terrestris* workers are all female, sex-specific effects could not be evaluated in this study.

### Method details

#### Hypoxia tolerance detection

The method for measuring oxygen concentrations was adapted from previous studies.^43^ Hypoxia tolerance was assessed by determining the stupor rate. Specifically, bumblebees were exposed to five different *P*o_2_ levels, namely 1.2, 1.6, 2.0, 2.4 and 2.8 kPa for 6 h. Bumblebee workers were placed inside a cage (10 × 15 × 15 cm), which was then positioned in a hypoxic chamber (FLYDWC-50; Fenglei Co., Ltd., China). The chamber was equipped with an automatic control system to regulate ambient temperature and airflow. To achieve the desired *P*o_2_ levels, a mixture of air and pure nitrogen was introduced into the chamber and adjusted precisely. The control group was maintained at a *P*o_2_ of 21 kPa, with at least three biological replicates of ten bumblebees for each assay.

#### Flight assay

The flight activity of bumblebees was quantified using a modified computer-aided flight mill system. The flight mill featured an arm 7 cm in length, resulting in a flight circumference of 0.44 m. The upper free-rotating body of the flight mill consisted of a 14 cm plastic pole, which supported the mill arms and served as a bearing on the pivot pin. Bumblebees were tethered to the end of the lightweight arm using a fine thread, allowing them to fly along a trajectory determined by the motion of the arm. The flight mill was connected to a computer that recorded electrical signals triggered by arm rotation, which interrupted an infrared beam.

For each bumblebee, flight parameters, including total flight distance, flight duration, and mean flight velocity, were recorded. A minimum of 10 bumblebees were randomly assigned to both experimental and control groups. Each bumblebee was tethered and subjected to a 60-min flight assay. To maintain continuous flight, a 2-s air puff (1.5 m/s) was applied at 30-s intervals whenever flight was interrupted. All assays were performed on individual bees Individuals with flight durations consistently less than 0.2 h were excluded from the analysis.

#### Respiration measurement

Resting metabolic rates were measured using a flow-through respirometry system (Sable Systems International, Las Vegas, NV, USA). To assess the effect of hypoxia on metabolic rate, worker bees were exposed to five oxygen partial pressures (*P*o_2_): 1.6, 2.4, 6.0, 12.0, and 21.0 kPa (*n* = 7 per treatment). Oxygen partial pressures were controlled by introducing a mixture of air and pure nitrogen at the system inlet instead of ambient air. Individuals were placed in the chamber under the target *P*o_2_ for a 30-min acclimation period prior to measurement.

Measurements were conducted in a 14 mL open-flow chamber at a constant airflow rate of 150 mL min^−1^, using a modified method.^92^^,^^93^ To ensure stable baseline conditions, incoming air was scrubbed of water vapor using Drierite (Sigma Aldrich, St. Louis, MO, USA). The rate of CO_2_ production was determined using the Sable Systems ExpeData analysis software (version 1.9.27). Metabolic rate was calculated as the rate of CO_2_ production per gram of body mass (mL min^−1^ g^−1^). All measurements were performed between 9:00 and 12:00 each day to control for potential circadian effects.

#### Mitochondrial morphology assay

Flight muscles from bumblebees in each treatment group were rapidly dissected and initially fixed in ice-cold 4% paraformaldehyde for 30 min. The muscle tissues were then cut into ∼1 mm^3^ blocks and post-fixed in 2.5% glutaraldehyde for 24 h. After fixation, samples were rinsed in 0.1 M phosphate-buffered saline (PBS), dehydrated through a graded ethanol series, and embedded in Epon epoxy resin. Polymerization was carried out by gradual heating for 24 h. Semi-thin sections (0.6 μm) were cut and stained with toluidine blue for localization under a light microscope. Ultrathin sections were subsequently double-stained with uranyl acetate and lead citrate. Finally, high-resolution ultrastructural images were obtained using a JEM-100SX transmission electron microscope (JEOL, Japan).

#### Carbohydrate metabolism measurements

Pyruvate (PA) levels were quantified using a commercial kit (Solarbio, BC2205; microplate method). ATP concentrations were measured with an ATP assay kit (Boxbio, AKOP004M; microplate method). Glycogen was assessed using a glycogen assay kit (Nanjing Jiancheng, A043-1-1; colorimetric method). Glucose levels were determined with a glucose assay kit (Nanjing Jiancheng, A154-1-1; microplate method). Trehalose content was measured using a trehalose assay kit (Nanjing Jiancheng, A149-1-1; anthrone colorimetric method). Total glutathione (GSH) and oxidized glutathione (GSSG) were quantified using a glutathione assay kit (Nanjing Jiancheng, A061-1-1; microplate method). All assays were performed strictly according to the manufacturers' protocols to ensure reproducibility.

#### RNA sequencing and expression data processing

Thoracic muscle tissues from worker bumblebees exposed to hypoxia treatments at 1.6 kPa and 21 kPa *P*o_2_ were dissected and immediately frozen in liquid nitrogen. Three independent replicates, each consisting of three worker bumblebees, were prepared for each treatment. Total RNA was extracted following the manufacturer’s protocol using the Eastep Super Total RNA Extraction Kit (Promega, USA). Subsequently, cDNA libraries were constructed according to Illumina’s protocols. After sequencing, raw reads were filtered to remove adaptor sequences, contaminants, and low-quality reads. Gene expression levels were quantified using fragments per kilobase of transcript per million mapped reads (FPKM). DEGs were identified using DESeq2 software, with a Benjamini-Hochberg adjusted *p* value threshold of 0.05. Functional enrichment analysis was performed using ClusterProfiler 4.0, and statistical significance was evaluated using Fisher’s Exact test to identify overrepresented terms.

#### RNA extraction and qPCR

Total RNA was extracted using the Eastep Super Total RNA Extraction Kit (Promega, USA). First-strand cDNA was synthesized from 1 μg of total RNA using M-MLV Reverse Transcriptase and oligo (dT) primer (Promega) according to the manufacturer’s instructions. cDNA transcripts were quantified using an SYBR Green kit on a LightCycler 480 instrument, also following the manufacturer’s instructions (Roche, USA). The relative expression of mRNA was analyzed using the 2^-ΔΔCT^ method. Dissociation curves were determined for each gene to confirm the presence of unique amplification products. At least three biological replicates were used for each treatment. Primers for qRT-PCR were designed using Primer 5.0 software and *UBI* was used as a reference gene to normalize the data. The primer sequences are provided in Table S1.

#### Multiple sequence alignment and phylogenetic analysis

To validate the categorization of 5-HT-related proteins, phylogenetic analysis was conducted using the full-length amino acid sequences of representative proteins from various species retrieved from the GenBank database. The amino acid sequences were aligned using the ClustalW program, integrated into MEGA 11. Phylogenetic trees were constructed using the neighbor-joining method with 1000 bootstrap replications. Motifs were analyzed using the MEME suite server (https://meme-suite.org/meme/) and conserved domains and transmembrane domains were analyzed using the NCBI and TMHMM server v. 2.0 (http://www.cbs.dtu.dk/services/TMHMM/), respectively. The resulting figures were refined using TBtools and AI software.

#### Antagonist supplementation

To evaluate the role of *5-HT*_*1A*_ in flight performance, bumblebees were treated with WAY-100635, a potent and selective *5-HT*_*1A*_ antagonist.^94^^,^^95^^,^^96^ A 25 mg/mL stock solution of WAY-100635 (CAS No.: 162760-96-5, MCE, USA) was prepared by dissolving the antagonist in dimethyl sulfoxide (DMSO). Bumblebee workers were randomly assigned to four groups: normoxic (0 + DMSO group), normoxic + antagonist treatment (WAY-100635+ DMSO group), hypoxia-exposed (0 + DMSO group), and hypoxia-exposed + antagonist treatment (WAY-100635+ DMSO group). Injections were administered with a working solution of 2.5 mg/mL at a volume of 5 μL. Tethered flight experiments were conducted under fasting conditions 8 h post-injection.

### Quantification and statistical analysis

Statistical analyses were performed using SPSS version 15.0 (SPSS Inc., Chicago, IL, USA). Data are presented as the means ± s.e.m. For all flight performance assays and biochemical comparisons between two groups were made using a two-tailed unpaired Student’s *t* test. For comparisons among multiple groups, a one-way ANOVA followed by Tukey’s test was applied. The threshold for statistical significance was set at *p* < 0.05.
