# Supplementary material for: Oxygen-dependent regulation of serotonin receptor 5-HT1A in hypoxic response and flight performance in bumblebees, Bombus terrestris
Source: iScience. 2025 Nov 25;28(12):114211. doi: 10.1016/j.isci.2025.114211 (PMC12690251; doi:10.1016/j.isci.2025.114211)
Supplement: Document S1. Figures S1 and S2 and Tables S1 and S2 [file mmc1.pdf]

iScience, Volume *28*

Supplemental information

Oxygen-dependent regulation of serotonin receptor *5-HT$_{1A}$* in hypoxic response and flight performance in bumblebees, *Bombus terrestris*

Chunyan Jiang, Panlong Meng, Xuexiao Du, Yingmin Sun, Xianliang Huang, and Bing Chen

Supplemental figures

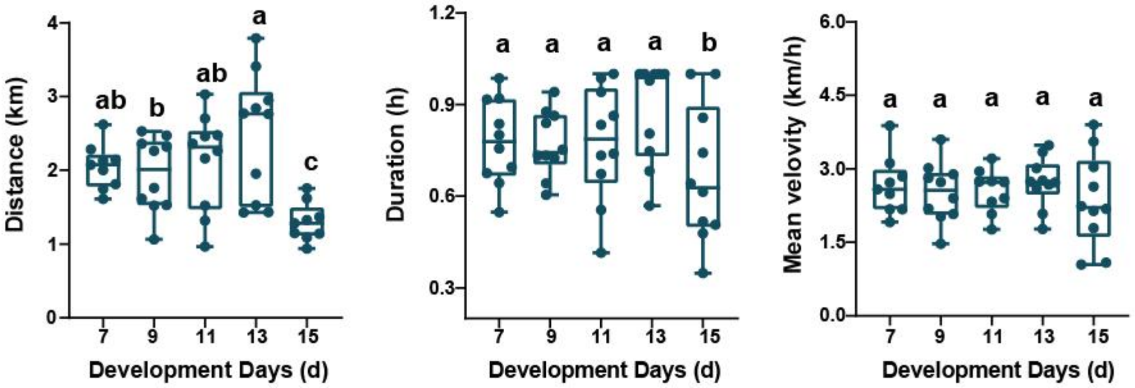

Figure S1. Flight performance of bumblebees at different developmental days (n = 10), Related to Figure 1. Data are presented as means (central lines) ± s.e.m (upper and lower bound of the bars). Statistical significance was determined using Student's *t*-tests. Distinct letters indicate statistically significant differences ($P < 0.05$), determined by one-way ANOVA.

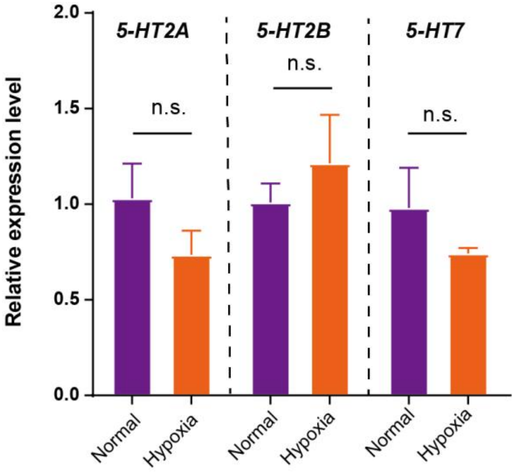

Figure S2 Expression responses of other 5-HT receptor genes to hypoxia in *B. terrestris* using qRT-PCR, Related to Figure 4. n = 3 biological replicates of three bumblebees for all assays.

Supplemental Tables

Table S1 PCR primer sequences for qPCR experiments, Related to STAR Methods

Primer name	Requence (5'-3')
UBI-PF	GGTATTTGGATGCCAGTGATTT
UBI-PR	ATGGGCATTTCTACCCCTTTTA
5-HT$_{1A}$-PF	CGAAATCTACAATCAGTGGC
5-HT$_{1A}$-PR	AGCAGTGCAGCAGAGGAC
GABAB1-qF	GCTAATTTCGCTTTCGTTGC
GABAB1-qR	AGCTCGTCGTTCTCGCTAAG
ccHa2-qF	TGTTGGTGTCTCCGTTTTCA
ccHa2-qR	TTCATCACGCTGGAGAACAG
mAchR-qF	ACCGTTGAGAAACCCATCAG
mAchR-qR	CCGTCTCCTCGTGGTACATT
mGlu-qF	CTGGCAGCGAGGTAAAGTTC
mGlu-qF	GCATGCCGAGATTGGTAGAT
5-HT$_{2A}$*-qF*	GCGTTCTTCGTGTTCGGTTC
5-HT$_{2A}$*-qR*	AACGAGCCTTCTGTCTGAGC
5-HT$_{2B}$*-qF*	GGAAGTACAGGTTTAGACAGACG
5-HT$_{2B}$*-qR*	CGATGTACCGGAAGTGTGCT
5-HT$_{7}$*-qF*	GATATTCGCCCTACACGCCA
5-HT$_{7}$*-qR*	CACAGCCACGCAGACTAGAA

Table S2 Detail information of 5-HT family genes, Related to Figure 4.

Gene name	ID	Gene name	ID
Pxu 5-HT$_{1}$	no. XP013180453	*H.S 5-HT*$_{1E}$	no.NP000856
Ms 5-HT$_{1A}$	no. ABI33826	*H.S 5-HT*$_{7A}$	no. NP000863
Ms 5-HT$_{1B}$	no. ABI33827	*H.S 5-HT*$_{7B}$	no. NP062874
Dm 5-HT$_{1A}$	no. CAA77570	*H.S 5-HT*$_{2B}$	no. NP000858
Dm 5-HT$_{1B}$	no. CAA7757	*H.S 5-HT*$_{2A}$	no. NP000612
Dm 5-HT$_{7}$	no. NP524599	*H.S 5-HT*$_{2C}$	no. NP000859
Dm 5-HT$_{2A}$	no. NP730859	*Bt 5-HT*$_{1A}$	no. XP003400301.
Dm 5-HT$_{2B}$	no. NP649806	*Tc 5-HT*$_{1A}$	no. XP967449
Pea 5-HT$_{1}$	no. CAX65666	*Tc 5-HT*$_{1B}$	no. XP972856
Am 5-HT$_{1A}$	no. CBI75449	*Tc 5-HT*$_{2B}$	no. EFA04642
Am 5-HT$_{7}$	no. CAJ28210	*Tc 5-HT*$_{7}$	no. XP966577
Am 5-HT$_{2A}$	no. NP001189389	*H.S 5-HT*$_{1A}$	no. NP000515
Am 5-HT$_{2B}$	no. NP001191178	*H.S 5-HT*$_{1B}$	no. NP000854
Bm 5-HT$_{1}$	no. CAA64862	*H.S 5-HT*$_{1D}$	no. NP000855
Aae 5-HT$_{7}$	no. AAG49292	*Gb 5-HT*$_{2A}$	no. BAJ83481
Ap 5-HT$_{1A}$	no. ABY85410	*Gb 5-HT*$_{1A}$	no. BAJ83479
Ap 5-HT$_{1B}$	no. ABY85411	*Gb 5-HT*$_{7}$	no. BAJ83482
Bt 5-HT$_{2A}$ (100648327)	no. XP_003397807.1	*Bt 5-HT*$_{2AB}$ (100645334)	no. XP_048265438.1
Bt 5-HT$_{7}$ (100645855)	no. XP_048262524.1		
